# Evaluation of the antimicrobial efficacy of the nanoshield barrier on potentially contaminated materials used by students in dental clinics: a cross-sectional study

**DOI:** 10.3389/froh.2026.1851913

**Published:** 2026-06-23

**Authors:** Vijay B. Desai, Raghavendra M. Shetty, Salem Abu Fanas, Sudhir Varma, Sunaina Shetty Yadadi

**Affiliations:** 1 Department of Clinical Sciences, College of Dentistry, Ajman University, Ajman, United Arab Emirates; 2 Center of Medical and Bio-allied Health Sciences Research, Ajman University, Ajman, United Arab Emirates; 3 Department of Pedodontia and Preventive Dentistry, Sharad Pawar Dental College and Hospital, Datta Meghe Institute of Higher Education and Research (Deemed to be University), Wardha, India; 4 Department of Restorative Dentistry, College of Dental Medicine, University of Sharjah, Sharjah, United Arab Emirates; 5 Microbiota Research Group Member, Research Institute of Medical and Health Sciences, University of Sharjah, Sharjah, United Arab Emirates

**Keywords:** antimicrobial surface barrier, bacterial contamination, copper nanoparticles, cross-infection control, dental clinic contamination, healthcare-associated infections, mobile phones, nanoshield technology

## Abstract

**Background:**

Environmental surfaces and devices routinely used in dental clinics are frequently exposed to microbial contamination from aerosols, droplets, and hand contact, posing a potential risk for cross-infection among patients and healthcare personnel.

**Aim:**

This study aimed to evaluate the antimicrobial efficacy of the Nano shield barrier (Nanoveu) on mobile phones, keyboards, computer mouse, and case history files commonly used by dental students in a clinical setting.

**Methodology:**

Nanoveu (Nanoveu Ltd), based on nano-shield technology, is a copper-based nanoparticle antimicrobial film. A total of 1000 items (250 mobile phones, 250 keyboards, 250 computer mouse, and 250 case history files) were included in the study. Each device type was equally divided into two halves. One-half was covered with Nanoveu (intervention group) and the other half was covered with a conventional plastic barrier (control group). After two weeks of exposure during dental procedures, swab samples were collected using HiCulture transport swabs, cultured on 5% sheep blood agar and Mac-Conkey agar, and incubated aerobically at 37°C for 24 hours. Data were analyzed using the Kruskal-Wallis and Mann-Whitney U tests through IBM-SPSS.

**Results:**

Nanoveu reduced TBC by approximately 63% on mobile phones (596.96 vs 218.96), 70% on case history files (455.04 vs 135.52), 70% on keyboards (797.92 vs 240.16), and 68% on computer mouse (630.4 vs 199.28) compared to the conventional plastic barrier.

**Conclusion:**

Nanoveu demonstrated significant antimicrobial efficacy against bacterial contamination on commonly used dental clinic surfaces, supporting its application as a protective barrier in clinical practice.

## 1 Introduction

Ensuring microbial hygiene standards is essential for maintaining good health. One of the main reasons for health issues may be a lack of understanding about where germs can originate. It is a fact that contact between hands and other items spreads 80% of illnesses (1). Cross-infection refers to the spread of infection between humans and non-living things, such as medical equipment and diagnostic tools used in clinics. Human-mediated infections include those spread by hospital staff, patients, or their guardians. Numerous dangerous bacteria are present on inanimate objects, acting as reservoirs for nosocomial infections and perhaps the leading cause of cross-contamination, which further spreads diseases (2). It is widely recognized that microbes from the surrounding environment can reside on inanimate objects. Gram-positive cocci predominate, although spore-forming rods and Gram-negative bacteria can also spread via computer keyboards, mobile phones, and case history file surfaces. Many pathogenic agents can persist on surfaces for long periods if they are not eliminated via sterilization or disinfection (1, 2). In dentistry, there 's a higher chance of airborne microbe contamination, along with a direct risk of disease transmission through hands or other tools. Bacterial aerosols produced by high-speed dental handpieces with water supply are frequently found in dental setups and can settle over great distances. Several infections may spread through aerosols and spatter created during dental operations (3). Potentially hazardous infections, such as multidrug-resistant organisms, can occur in therapeutic settings. Dry surface biofilms, which are dynamic microbial communities found on dry surfaces, play a role in prolonging bacterial survival in the environment (4). In a dental setting, every microbe that enters the bloodstream or colonizes the oral cavity has the potential to spread. There is a significant risk of disease transmission from high-touch surfaces, such as computers, phones, and case history files, due to aerosols, gloved hands, and instrument contact. They act as a transmissible pool for microbiological pathogens, which further links them to nosocomial illnesses (1, 4). By severing the chain of transmission, which requires adherence to surface cleaning procedures to the fullest degree, it is possible to stop the spread of potentially dangerous germs in the dental context. According to the Centers for Disease Control and Prevention (CDC) standards (3, 4), the right way to maintain surfaces in a dental operatory is to clean, disinfect, and use surface barriers.

A novel tactic to stop cross-contamination is barrier protection, which involves applying water-resistant, impenetrable barriers to frequently touched surfaces (4, 5). The barriers utilizing nanotechnology-based techniques facilitate the development of nanoscale surfaces that can decrease bacterial adhesion by causing irreversible damage to DNA and cell membranes. These structures also have long plasma half-lives and, because of their high surface-to-volume ratio, make it easier to target things (5). Nanoveu, a novel antimicrobial film with nano-shield technology, is available in the market and has been reported to exhibit broad-spectrum antimicrobial activity attributed to the generation of copper ions and reactive oxygen species (ROS). It produces electrically charged copper ions and reactive oxygen species, directly attaches to viruses and bacteria, alters their structure, and exhibits antimicrobial activity.

In areas where infections are common, it is imperative to implement techniques that reduce microbial populations, as the unchecked and rapid growth of potentially hazardous bacteria can have negative consequences. The level of contamination of materials in dental clinics has not been precisely proven, especially those based on nanoshield technology. There is a paucity of literature on how to decontaminate commonly exposed surfaces in a dental clinic. This restriction may arise because of the lack of well-defined cleaning requirements for devices in dental workplaces. Despite growing evidence of surface contamination in dental settings, there is a paucity of data on the antimicrobial efficacy of nanoshield technology-based products on commonly exposed dental clinic surfaces. Hence, the current study aimed to evaluate the bacterial contamination and antimicrobial efficacy of nano-shield barrier (Nanoveu) on mobile phones, keyboards, mouse, and case history files used by students in the dental clinic.

## 2 Materials and methods

### 2.1 Study design and ethical considerations

Approval was obtained from the Ethics and Research Committee of Ajman University (Ref No.: D-F-H-11-Oct) before the commencement of the study. This cross-sectional comparative study was conducted during active clinical sessions at the College of Dentistry, Ajman University, Ajman, UAE, from December 2023 to April 2025, following good laboratory practice guidelines. Furthermore, it is verifiable that the study was carried out in full accordance with the Declaration of Helsinki of the World Medical Association.

### 2.2 Sample size, sampling, and sample collection

The sample size for the study was calculated using the G*Power program (Version 3.1.9.4). The sample size was estimated to be 250 in each group at a power of 0.95 and an alpha error of 0.05. The simple random sampling technique was employed, and a total of 1000 items (250 mobile phones, 250 keyboards, 250 computer mouse, and 250 case history files were included in the study. Each device type was equally divided into two halves. One-half was covered with Nanoveu (intervention group) and the other half was covered with a conventional plastic barrier (control group). The steps followed were: (1) Random selection of 250 units per device category; (2) Application of disinfection protocol per institutional guidelines; (3) Random allocation of one-half of each unit to either Nanoveu or conventional barrier; (4) Two-week exposure period; (5) Swab sample collection from each surface of both intervention and control. According to the disinfection protocol of the College of Dentistry, Ajman University, keyboards and mouse were disinfected with a hospital tuberculocidal disinfectant before the treatment sessions, while the mobile phones and case history files were sterilized with the disinfectant wipes before the patients were allotted to the dental students. The conventional plastic barrier used as a control was a standard polyethylene (PE) low-density plastic sheet, commonly used in dental infection control as a disposable surface cover. This barrier serves as a passive physical shield with no intrinsic antimicrobial properties, in contrast to the copper-nanoparticle-embedded Nanoveu film.

After two weeks, samples were obtained from the investigated surfaces using disposable sterile cotton swabs pre-moistened with sterile saline. The swabs were thoroughly rotated over the front and back surfaces of the mobile phones, keyboards, mouse, and case history files. Separate samples were collected from the intervention and control groups for microbiological analysis.

#### 2.2.1 Mechanism of action of nanoshield barrier (Nanoveu)

**Figure 1:**
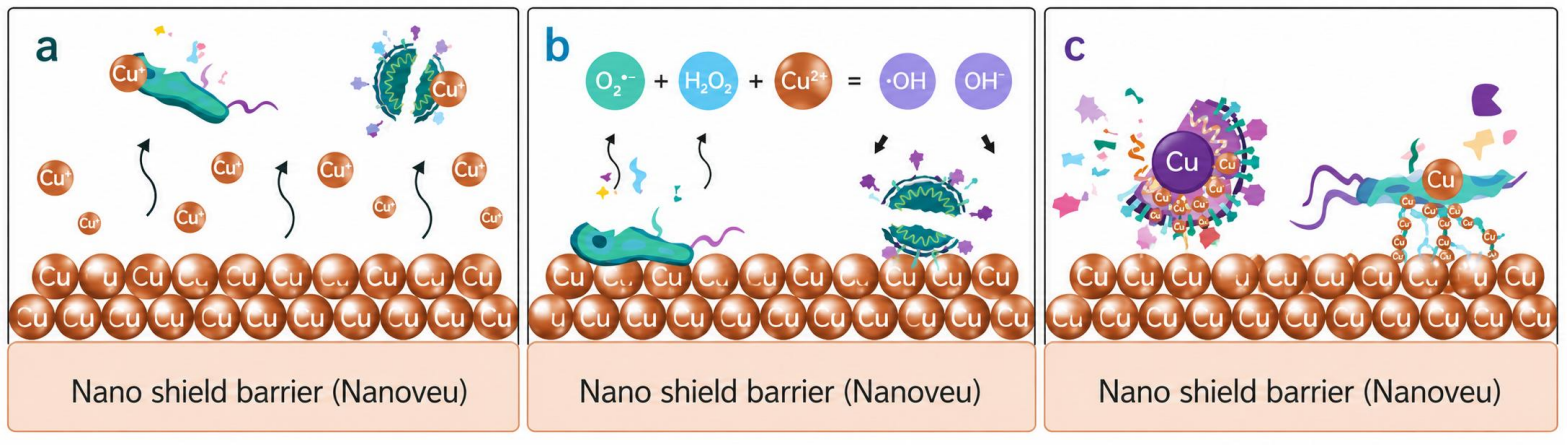
Mechanism of action of nanoshield barrier (Nanoveu): **(a)** copper ions, **(b)** reactive oxygen species (ROS), and **(c)** direct contact.

The nano-shield barrier works on copper nanotechnology. Nanoshield produces electrically charged copper ions that attach to viruses and bacteria, altering their structure and stopping their functioning (Figure 1a). Nanoshield reacts with molecules produced by bacteria (H_2_O_2_ and O_2_•^–^) to form a chemical substance called reactive oxygen species. ROS damages both proteins and nucleic acids in viruses and bacteria, providing antibacterial and antiviral effects (Figure 1b). All viruses are surrounded by a protective protein layer that holds the infectious components. For viruses to spread, this layer must remain intact. Nano-sheild copper particles were rapidly absorbed into this protein layer, bursting their protective walls (Figure 1c).

### 2.3 Isolation

Samples were collected using the HIculture™ transport swab MS5259 for environmental monitoring. The long screw cap tube containing the sponge with saline was unscrewed. The flock-tip swab was inserted into the sponge to moisten its tip. The template was placed at the sampling surface and swabbed in the bidirection to achieve maximum uptake of the surface material. After sampling was completed, the swab was inserted into the second tube with buffered peptone water and broken at the neck by applying pressure diagonally. The samples were transported to the laboratory within 4 h under cool conditions for a microbiological assay, following these steps: (1) unscrew the long screw-cap tube of the HiCulture™ transport swab (MS5259); (2) insert the flock-tip swab into the sponge to moisten the tip; (3) place the template on the surface and swab bidirectionally; (4) insert the swab into the secondary tube containing buffered peptone water and break it at the neck; and (5) transport to the laboratory within 4 h under cool conditions.

### 2.4 Identification of isolates

The samples were streaked onto 5% sheep blood agar and MacConkey agar after inoculation. For 24 h, the agar plates were incubated aerobically at 37°C. The distinctive appearance of the colonies on the cultivation media, which included hemolysis and usual growth characteristics, was used for identification. MicroScan WalkAway equipment was used for identification and susceptibility testing. It measures the biochemical reactivity and antibiotic susceptibility of bacterial isolates using microtiter panels that include dry metabolic substrates and antibiotics. Both chromogenic and fast fluorescence panels can be read using this device.

### 2.5 Statistical analysis

The collected data were entered into MS Excel and analyzed using IBM-SPSS. The normality of the data distribution was determined using the Shapiro–Wilk test, and the data were found not to be normally distributed. Descriptive statistics were presented as frequencies with percentages for categorical variables and the mean ± standard deviation for continuous variables. Statistical tests, Kruskal–Wallis and Mann–Whitney *U* tests, were applied. Statistical significance was set at *P* ≤ 0.05. The Kruskal–Wallis test was used to compare microbial counts across the four device types (mobile phone, keyboard, mouse, and case history files). The Mann–Whitney *U* test was used for pairwise comparisons between the Nanoveu (intervention) and conventional barrier (control) groups for each device.

## 3 Results

All tested surfaces were contaminated with mixed growth of mainly Gram-positive and Gram-negative pathogenic and non-pathogenic bacterial isolates. The isolates found on the examined surfaces included mainly *Klebsiella*, *Enterococcus faecalis*, and *Staphylococcus aureus*. A higher TBC and greater *Klebsiell*a counts were frequently identified on all the surfaces. The mean TBC was highest for the mobile phones (282.8) and lowest for the case history files (210.44), while the mean growth of *Klebsiella* was highest for the keyboards (297.86) and lowest for the mobile phones (219.11), with statistically significant differences among them (*P* < 0.0001). *E. faecalis*, a Gram-positive commensal, was most identified on keyboards used in the dental clinic, both with (67.2 ± 269.544) and without shields (238.64 ± 924.731), and least discovered on the surfaces of the case history files, both with (1.04 ± 9.986) and without the application of shields (5.44 ± 43.506). The growth of *S. aureus* was negligible on case history files, while keyboards harbored a significant presence both with (4.8 ± 11.612) and without shields (14.64 ± 26.444). The degree of bacterial contamination on frequently exposed surfaces in a dental setting with and without the use of nano-shield technology as a disinfectant is shown as the mean and standard deviation in Table 1.

**Table 1:** Distribution of microbial contamination of frequently touched surfaces in dental settings with and without the application of nanoshield technology.

Device	Group	Microorganisms	Mean ± SD	*P* value
Phone	With shield	TBC	218.96 ± 196.394	0.042*
		*Klebsiella*	96.88 ± 137.702	
		*E. faecalis*	3.44 ± 26.092	
		*S. aureus*	0.64 ± 5.197	
	Without shield	TBC	596.96 ± 413.723	≤0.001**
		*Klebsiella*	317.52 ± 349.739	
		*E. faecalis*	6.8 ± 47.631	
		*S. aureus*	1.36 ± 15.205	
Files	With shield	TBC	135.52 ± 119.376	0.003*
		*Klebsiella*	136.64 ± 163.006	
		*E. faecalis*	1.04 ± 9.986	
		*S. aureus*	–	
	Without shield	TBC	455.04 ± 328.709	0.028*
		*Klebsiella*	451.04 ± 406.239	
		*E. faecalis*	5.44 ± 43.506	
		*S. aureus*	–	
Keyboard	With shield	TBC	240.16 ± 477.308	≤0.001**
		*Klebsiella*	143.52 ± 266.928	
		*E. faecalis*	67.2 ± 269.544	
		*S. aureus*	4.8 ± 11.612	
	Without shield	TBC	797.92 ± 1,483.143	0.037*
		*Klebsiella*	441.04 ± 695.905	
		*E. faecalis*	238.64 ± 924.731	
		*S. aureus*	14.64 ± 26.444	
Mouse	With shield	TBC	199.28 ± 374.989	0.089*
		*Klebsiella*	109.52 ± 256.357	
		*E. faecalis*	77.36 ± 264.612	
		*S. aureus*	3.28 ± 9.313	
	Without shield	TBC	630.4 ± 1,262.679	≤0.001**
		*Klebsiella*	341.52 ± 686.304	
		*E. faecalis*	171.12 ± 506.404	
		*S. aureus*	10.88 ± 25.464	

All values are expressed as the mean ± standard deviation (SD). The Kruskal–Wallis test is used as the statistical test; level of significance: *P ≤ 0.05 is considered statistically significant, and **P ≤ 0.001 is highly significant.

**Figure 2:**
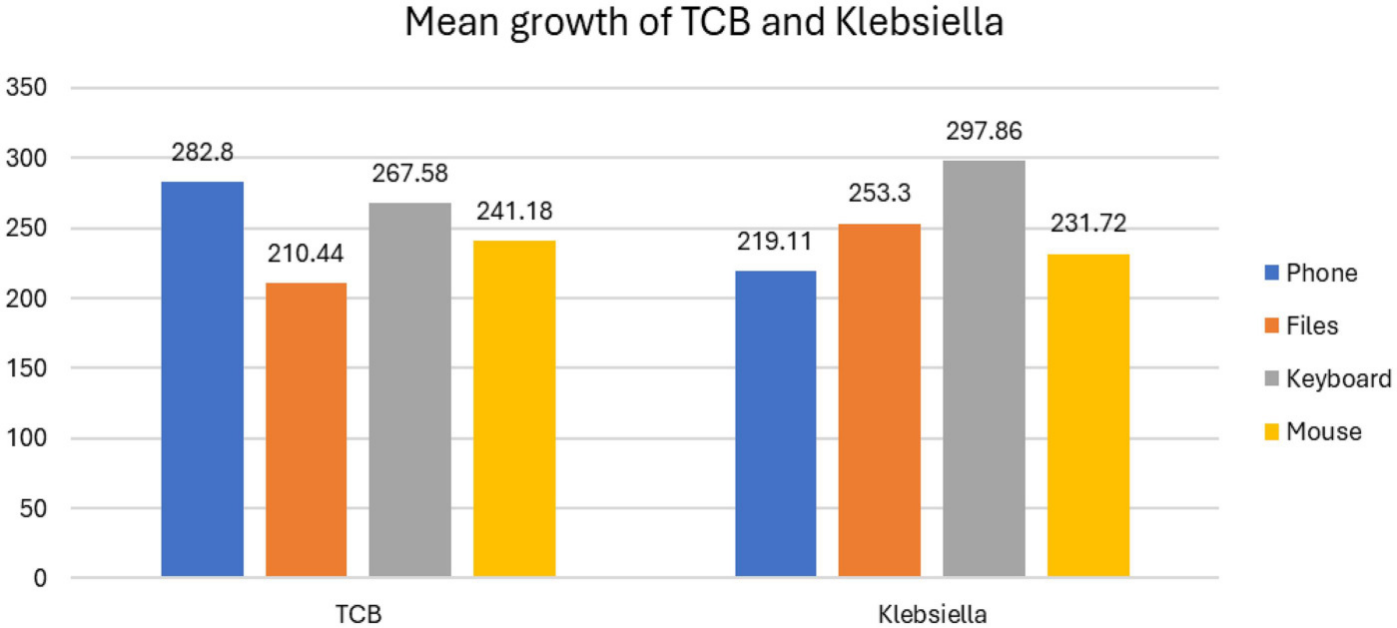
Proportions of test sites with the mean growth of TBC and *Klebsiella* on the respective surfaces.

Figure 2 shows a comparative analysis depicting the mean growth of *Klebsiella* and the total bacterial count. The mean rank of the microorganisms on all the surfaces, that is, mobile phones (89.23), keyboards (91.13), mouse (93.18), and case history files (87.68), treated with nanoshield was significantly less than that without a shield, which indicates that the efficacy of nano-shield in terms of bacterial reduction was higher in comparison to surfaces treated without a shield. TBC was highly significant in cases involving mobile phones and mouse when a shield was not applied (*P* < 0.001). In terms of computer contamination, keyboards were significantly more contaminated than the computer mouse. The mean ranks of microorganisms with and without nano-shield technology on different surfaces are depicted in Table 2.

**Table 2:** Mean rank of microorganisms with and without nanoshield technology, projecting the efficacy of nanoshield technology on various surfaces.

Device	Group	Mean rank	Mann–Whitney *U*	*P*-value
Phone	With shield	89.23	3,279.0	<0.001**
	Without shield	161.77		
Files	With shield	87.68	3,085.5	<0.001**
	Without shield	163.32		
Keyboard	With shield	91.13	3,516.5	<0.001**
	Without shield	159.87		
Mouse	With shield	93.18	3,772.0	<0.001**
	Without shield	157.82		

All values are expressed as the mean ± standard deviation (SD). The Mann–Whitney *U* test is used as the statistical test; level of significance: *P ≤ 0.05 is considered statistically significant, and **P ≤ 0.001 is highly significant.

**Figure 3:**
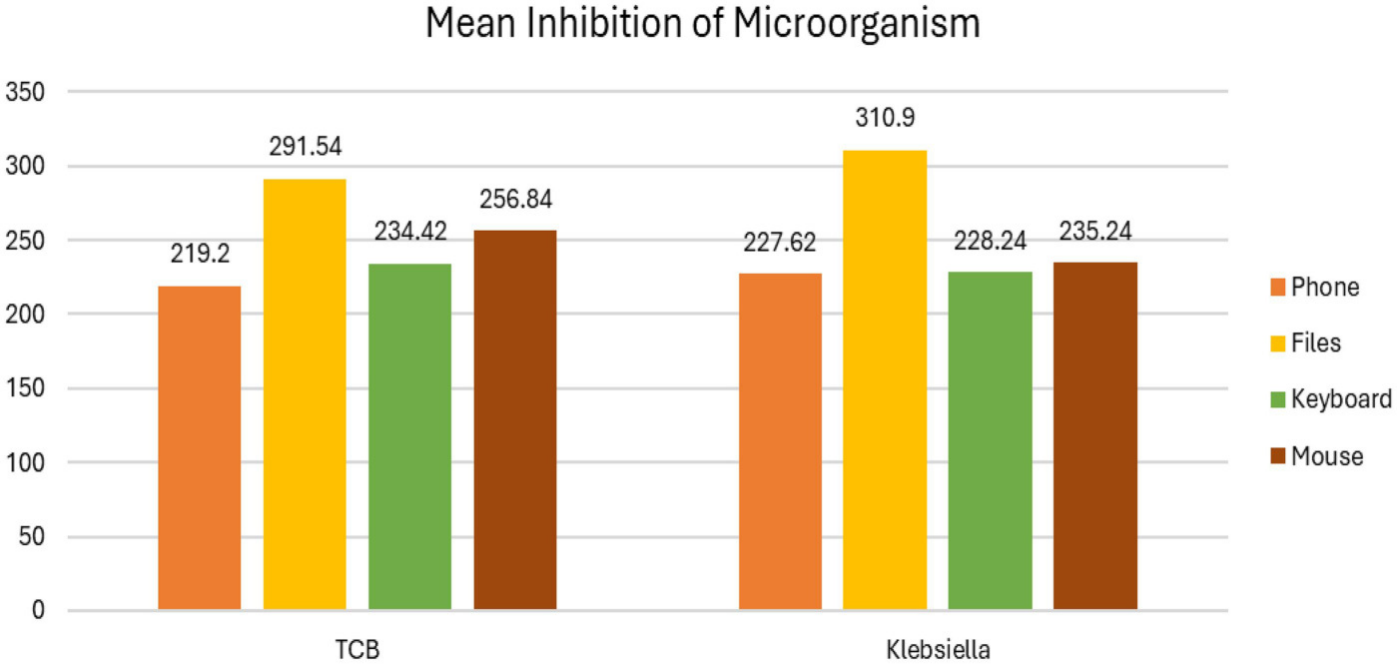
Proportions of test sites with mean inhibition of TBC and *Klebsiella* with shield on respective surfaces.

The mean inhibition of TBC (291.54) and *Klebsiella* (310.9) was best appreciated with case history files treated with the nano-shield antimicrobial barrier. The mean inhibition of TBC and *Klebsiella* with the shield on all surfaces examined is depicted in Figure 3.

## 4 Discussion

The bio-contamination of surfaces on various public goods and equipment is one of the primary sources of nosocomial illnesses and epidemics derived from the environment (3). The current study is the first of its kind to evaluate the antimicrobial effectiveness of Nanoveu, a nanoparticle-based disinfectant. The findings of the present study demonstrated that it was effective on all the surfaces tested compared to the conventional plastic barrier used as a control in the elimination of bacteria and inhibition of their growth in the medium. The probable reason for this could be due to its innovative triple mechanism of action. Their high surface-to-volume ratio renders them extremely reactive antibacterial materials. Nanoshield also reacts with molecules produced by bacteria to form a chemical substance called ROS. ROS damages both proteins and nucleic acids in viruses and bacteria, providing antibacterial and antiviral effects (4). Since Nanoveu is made of copper-based nanoparticles, its antibacterial activity relies on the release of Cu^2+^ ions, which can penetrate bacterial cells, damage their membranes, and interfere with their ability to produce enzymes, ultimately leading to bacterial death (5). Unfortunately, there are currently no definite guidelines for the disinfection of inanimate objects that are frequently exposed to contamination in dental clinics; hence, this study could serve as a pioneer in establishing thorough protocols and further exploring the scope of nanoparticle-based disinfectants. In addition to acting as a reservoir for pathogens in the infectious chain, contaminated surfaces also act as fomites, spreading infectious organisms from inanimate to living things through hand-to-hand contact (5). A study found that illnesses, such as tuberculosis, staphylococcal infections, conjunctivitis, viral infections, and other skin infections, have been linked to bacteria found in dental aerosols. Therefore, maintaining basic hygiene is essential for a healthy living environment (6). A legitimate issue is the possibility of contamination by potential pathogens. Multidrug-resistant Gram-positive organisms, including *S. aureus* and enterococcal species, are the source of nosocomial infections, which are becoming an increasing concern in many healthcare facilities (7). The current study aimed to evaluate the antimicrobial efficacy of a novel nanoshield technology-based disinfectant, Nanoveu, on frequently exposed surfaces in a dental clinic, that is, mobile phones, keyboards, case history files, and computer mouse, while also attempting to isolate possible bacterial species inhabiting these surfaces.

In the current investigation, a mixed flora of Gram-positive and Gram-negative bacteria was found, with a greater total bacterial count and a higher incidence of *Klebsiella*. As commensals and epiphytes, these bacteria are components of the physiological microbiota of the skin and mucous membranes. Microorganisms grew more on keyboards and cell phones, which corresponds to the findings of Ulger et al. (8) and Soto et al. (9), which demonstrate the presence of several bacteria on mobile phones. They found that mobile phones commonly become contaminated through hands, bags, cases, pockets, as well as exposure to the environment and food residue. The increased temperature and humidity produced by a mobile phone facilitate the growth of germs and the formation of biofilms on the device surface. The bacteria in the biofilm may continue to be infectious for a few weeks, depending on the surrounding circumstances (10, 11). The study's conclusions were consistent with the research conducted by Al-Ghamdi et al., which investigated the presence of germs on mousepads, shopping cart handles, elevator buttons, and computer keyboards. These outcomes are predicted because human hands and fingers are common sites of microbial transmission (12). Koscova et al. evaluated the presence of microbes on computer keyboards and cell phones. *S. aureus* (20%) was found at the lowest concentration on the mobile phone surfaces, which is in agreement with the findings of the current study (1). Bhat et al. reported that *S. aureus*, *Escherichia coli*, *Klebsiella pneumoniae*, *Acinetobacter* spp., and *Pseudomonas aeruginosa* cause considerable (99%) contamination of mobile phones (13). Movahhed et al. found that dental school computers contained harmful microorganisms, such as *Klebsiella* and *S. aureus* (14). *S. aureus* was the least common organism found in the current study, and TBC was significantly higher, with *Klebsiella* being the most common organism present. Fard et al. concluded in their study that of 240 cultured mobiles, the growth was majorly polymicrobial (74%), while the most recurrently cultured organisms were *S. aureus*, *E. coli*, *E. feacalis*, and *Pseudomonas* (15). In a study conducted by Trivedi et al., *S. aureus*, *K. pneumonia*, and *Enterococcus* were found to be present on 46.66% of cell phones of hospital staff members. In contrast, our study found that *Klebsiella* was the most frequently isolated organism, with *Staphylococcus epidermis* accounting for 40% of cases (16). According to Scott and Bloomfield (2008), a sizable fraction of organisms that may be recovered on an agar surface can be transferred when contaminated surfaces come into even brief contact with fingers or an inanimate surface (17). Mobile phones and computer keyboards are among the most frequently shared and touched surfaces. In healthcare settings, these devices can act as reservoirs for microorganisms due to frequent handling, inadequate cleaning practices, and concerns regarding potential damage during disinfection (18). The superior efficacy of Nanoveu compared to conventional plastic barriers in this study is likely attributable to its active, copper-based antimicrobial mechanism. Cu^2+^ ions released from copper nanoparticles penetrate bacterial cell membranes and disrupt enzymatic activity while simultaneously generating ROS that damage nucleic acids and proteins. Passive plastic barriers lack this active mechanism and act only as physical shields without inherent biocidal properties.

A limitation of this study was that, in addition to identifying the specific microorganisms, the colony-forming units were not assessed to provide a thorough understanding of the bacterial contamination on the exposed surfaces. Further research is required to determine the effectiveness of regularly applying disinfectants based on nanoshield technology in lowering the microbiological contamination on frequently touched surfaces in dental settings. Implementing an infection control program is the need of the hour to stop the spread of infections in hospital settings. These findings suggest that increasing the practice rate of surface disinfection and educating dental hygiene students about its value in dental clinics are essential. Regarding biosafety, copper nanoparticles at concentrations effective against microbial cells have been shown in vitro to exploit differences in membrane composition and surface charge between prokaryotic and eukaryotic cells. Bacterial cell membranes, which generally lack cholesterol and possess a net negative surface charge, are more susceptible to Cu^2+^ ion interaction and membrane disruption than mammalian cell membranes. Nevertheless, cytotoxic effects on mammalian cells have been reported at higher CuNP concentrations, indicating that their therapeutic window remains concentration dependent (19, 20). As Nanoveu is applied as a surface film rather than a dispersed agent, direct systemic exposure to mammalian cells under normal conditions is considered negligible. However, formal biocompatibility studies under ISO 10993 standards are recommended to fully characterize the safety profile of Nanoveu in clinical environments.

## 5 Conclusions

Mobile phones showed the highest total bacterial count among all other objects evaluated. However, the commercially available antimicrobial film, Nanoveu, based on nanoshield technology, can reduce the presence of microorganisms on devices used regularly in dental clinics. It proves to be an effective advanced protective barrier, delivering consistent passive disinfection and ensuring that the surfaces are well-guarded against pathogens. Hence, monitoring systems for effective infection management are necessary to minimize cross-infection, establish effective preventive strategies for nosocomial infections, and promote a safer environment in dental clinics. Within the limitations of this cross-sectional study, Nanoveu demonstrated a significant reduction in bacterial contamination across all tested dental clinic surfaces compared to conventional plastic barriers, with mean TBC reductions ranging from 63% to 70%. These findings suggest that nanoshield technology may serve as a promising adjunctive passive disinfection strategy in dental clinical settings. Further longitudinal and multicenter studies are recommended to validate these findings and establish standardized protocols.

## Data Availability

The raw data supporting the conclusions of this article will be made available by the authors without undue reservation.

## References

[1] Koscova J Hurnikova Z Pistl J. Degree of bacterial contamination of mobile phone and computer keyboard surfaces and efficacy of disinfection with chlorhexidine digluconate and triclosan to its reduction. Int J Environ Res Public Health. (2018) 15(10):2238. 10.3390/ijerph15102238 30322055 PMC6210060

[2] Chatterjee S Saigal S Bhargava A Shankar D Khan AM Khan SF. Hidden reservoirs of pathogens in dental settings. Bioinformation. (2021) 17(1):73–9. 10.6026/97320630017073 34393421 PMC8340715

[3] Sattar SA. Survival of microorganisms on animate and inanimate surfaces and their disinfection. In: Rutala WA, editor. Disinfection, Sterilisation and Antisepsis: Principles and Practices in Healthcare Facilities. Washington, DC, USA: Association for Professionals in Infection Control and Epidemiology, Inc. (2001). p. 195–205.

[4] Vyprynyuk K. Best practices for surface disinfection (2023).

[5] Yılmaz GE Göktürk I Ovezova M Yılmaz F Kılıç S Denizli A. Antimicrobial nanomaterials: a review. Hygiene. (2023) 3(3):269–90. 10.3390/hygiene3030020

[6] Kohn WG Collins AS Cleveland JL Harte JA Eklund KJ Malvitz DM. Guidelines for infection control in dental health care settings—2003. MMWR Recomm Rep. (2003) 52(RR-17):1–61. PMID: 14685139 14685139

[7] Prathibha P Mohanlal B Sura Ali Ahmed Fouad AB. Is cellular phone a source of infection? A hospital based study among dentists in Ajman and Sharjah, UAE (2012).

[8] Ulger F Esen S Dilek A Yanik K Gunaydin M Leblebicioglu H. Are we aware how contaminated our mobile phones with nosocomial pathogens? Annals Clin Microbiol Antimicrob. (2009) 8:7. 10.1186/1476-0711-8-7 PMC2655280 19267892

[9] Soto RG Chu LG Goldman JM Rampil IJ Ruskin KJ. Communication in critical care environments, mobile telephones improve patient cares. Anaesth Analg. (2006) 102:534–41. 10.1213/01.ane.0000194506.79408.79 16428557

[10] Hassan AN Birt DM Frank JF. Behavior of Listeria monocytogenes in a Pseudomonas putida biofilm on a condensate-forming surface. J Food Prot. (2004) 67:322–7. 10.4315/0362-028X-67.2.322 14968965

[11] Kramer A Schwebke I Kampf G. How long do nosocomial pathogens persist on inanimate surfaces? A systematic review. BMC Infect Dis. (2006) 6:130. 10.1186/1471-2334-6-130 16914034 PMC1564025

[12] Al-Ghamdi AK Abdelmalek SMA Ashshi AM Faidah H Shukri H Jiman-Fatani AA. Bacterial contamination of computer keyboards and mice, elevator buttons and shopping carts. Afr J Microbiol Res. (2011) 5(23):3998–4003. 10.5897/AJMR11.770

[13] Bhat SS Hegde SK Salian S. Potential of mobile phones to serve as a reservoir in spread of nosocomial pathogens. Online J Health Allied Sci. (2011) 10:1–3.

[14] Movahhed T Dehghani M Ghoddusi T. Evaluation of microbial contamination of mobile phones and computer mice and keyboards in a dental school. J Dent Mater Tech. (2018) 7(2):78–82.

[15] Fard RH Fard RH Moradi M Hashemipour MA. Evaluation of the cell phone microbial contamination in dental and engineering schools: effect of antibacterial spray. J Epidemiol Glob Health. (2018) 8(3–4):143. 10.1016/j.jegh.2017.10.004 30864755 PMC7377557

[16] Trivedi HR Desai KJ Trivedi LP Malek SS Javdekar TB. Role of mobile phone in spreading hospital acquired infection: a study in different group of health care workers. Natl J Integr Res Med. (2011) 2:61–6.

[17] Scott E Bloomfield SF. The survival and transfer of microbial contamination via cloths, hands and utensils. J Appl Microbiol (2008) 68:271–8. 10.1111/j.1365-2672.1990.tb02574.x 2111304

[18] Koscova J Hurnikova Z Pistl J. Degree of bacterial contamination of mobile phone and computer keyboard surfaces and efficacy of disinfection with chlorhexidine digluconate and triclosan to its reduction. Int J Environ Res Public Health. (2018) 15(10):2238. 10.3390/ijerph15102238 30322055 PMC6210060

[19] Ma X Zhou S Xu X Du Q. Copper-containing nanoparticles: mechanism of antimicrobial effect and application in dentistry—a narrative review. Front Surg. (2022) 9:905892. 10.3389/fsurg.2022.905892 35990090 PMC9388913

[20] Woźniak-Budych MJ Staszak K Staszak M. Copper and copper-based nanoparticles in medicine—perspectives and challenges. Molecules. (2023) 28(18):6687. 10.3390/molecules28186687 37764463 PMC10536384

